# Precision Medicine in Oncology: A Review of Multi-Tumor Actionable Molecular Targets with an Emphasis on Non-Small Cell Lung Cancer

**DOI:** 10.3390/jpm11060518

**Published:** 2021-06-05

**Authors:** Matthew K. Stein, Oluchukwu Oluoha, Kruti Patel, Ari VanderWalde

**Affiliations:** 1 Missouri Baptist Medical Center, Heartland Cancer Research, NCI Community Oncology Research Program, St. Louis, MO 63131, USA; Matthew.stein@bjc.org; 2 Division of Hematology and Oncology, University of Tennessee Health Science Center, Memphis, TN 38103, USA; ooluoha@westclinic.com (O.O.); kpatel@westclinic.com (K.P.); 3 West Cancer Center and Research Institute, Germantown, TN 38138, USA

**Keywords:** cancer, next-generation sequencing, targeted therapy, precision oncology, tumor-agnostic indications, solid tumors, tumor markers, FDA-approved therapeutics

## Abstract

Precision medicine is essential for the modern care of a patient with cancer. Comprehensive molecular profiling of the tumor itself is necessary to determine the presence or absence of certain targetable abnormalities or biomarkers. In particular, lung cancer is a disease for which targetable genomic alterations will soon guide therapy in the majority of cases. In this comprehensive review of solid tumor-based biomarkers, we describe the genomic alterations for which targeted agents have been approved by the United States Food and Drug Administration (FDA). While focusing on alterations leading to approvals in a tumor-agnostic fashion (MSI-h, TMB-h, *NTRK*) and on those alterations with approvals in multiple malignancies (*BRAF*, *ERBB2*, *RET*, *BRCA*, PD-L1), we also describe several biomarkers or indications that are likely to lead to an approved drug in the near future (e.g., KRAS G12C, PD-L1 amplification, HER2 overexpression in colon cancer, HER2 mutations in lung cancer). Finally, we detail the current landscape of additional actionable alterations (*EGFR*, *ALK*, *ROS1*, *MET*) in lung cancer, a biomarker-rich malignancy that has greatly benefitted from the precision oncology revolution.

## 1. Introduction

Precision medicine, defined as supplying the right treatment to the right patient at the right time, has become an essential element of cancer care, Taking advantage of novel technologies developed following sequencing of the human genome approximately twenty years ago, precision oncology leverages a tumor’s molecular features with available and novel therapeutics [1,2,3,4]. Prior to the advancement of comprehensive tumor profiling, successful implementation of a precision oncology approach included tyrosine kinase inhibitors (TKIs) imatinib for breakpoint cluster region-Abelson (*BCR-ABL)*-rearranged chronic myeloid leukemia [5] and trastuzumab for human epidermal growth factor 2 (HER2) immunohistochemistry (IHC) overexpressed or amplified breast cancer [6,7].

Currently, molecular profiling is available to comprehensively characterize a patient’s tumor within as few as two weeks and includes interrogation of anywhere from hundreds of genes to the whole exome for mutations, insertions, deletions or copy-number alterations via next-generation sequencing (NGS), gene fusions with RNA sequencing and various protein changes with IHC. The goal of such extensive testing is to unveil the genomic makeup of a patient’s tumor, which can inform the most effective therapeutic approach. Oftentimes, this must be coupled with the malignancy’s site-of-origin and histologic features; however, precision treatment strategies are increasingly being employed in a tissue-agnostic fashion through a growing list of pan-tumor United States Food and Drug Administration (FDA) approvals, clinical trials and off-label use when a molecular approach can be justified (e.g., through a tumor board consensus) [8].

In this review, we will outline progress in precision medicine in oncology, with an aim to summarize the current landscape of FDA-approved therapies based upon predictive molecular biomarkers in solid tumors, focusing on those markers that determine therapeutic options in more than one malignancy type. As will be seen, several pan-tumor and multi-tumor FDA approvals exist for both tumors with alterations expressing sensitivity to targeted agents (including TKIs and antibody-drug conjugates (ADCs)) as well as immune checkpoint inhibitors (ICIs). The full extent of molecular targets in cancer cannot be completely summarized in a single paper, and we will concentrate on those findings deemed DNA-based alterations by most NGS vendors.

We will conclude by switching from biomarker to malignancy, detailing present treatment options available for non-small cell lung cancer (NSCLC). NSCLC serves as a model for precision oncology where patients can benefit substantially from employment of molecular profiling.

## 2. Molecular Alterations with Approvals Regardless of Tumor-Site

To date, three molecular targets (microsatellite instability-high (MSI-H) or mismatch repair deficient (MSI-H/dMMR), neurotrophic tropomyosin-related kinase (*NTRK1/2/3*) fusions or high tumor mutational burden (TMB-H)), have led to four site- or tumor-agnostic approvals by the FDA. These biomarkers and drugs with corresponding FDA approvals are listed in the first part of Table 1.

### 2.1. MSI-h/dMMR

The first of these approvals occurred on 23 May 2017 when pembrolizumab, an ICI whose activity lies in the inhibition of programmed cell death protein 1 (PD-1), was approved for second-line or later treatment of metastatic or unresectable solid tumors in pediatric and adult patients found to be MSI-H/dMMR [9]. The basis of the approval was from 149 MSI-H/dMMR patients spanning five uncontrolled, multi-cohort, multicenter, single-arm clinical trials (KEYNOTE-012, -016, -028, -158 and -164) [9,48,49,50,51], where the overall response rate (ORR) was 40% (95% confidence interval (CI): 31.7, 47.9) and responses lasted for >6 months in 78% of responders. In this cohort, 90/149 (60%) of patients had colorectal cancer, for which prior treatment with a fluoropyrimidine, oxaliplatin and irinotecan was required; 14 other tumor types were evaluated leading up to the 2017 FDA approval.

Several other ICI agents are FDA approved for MSI-H/dMMR colorectal cancer patients. These include nivolumab following treatment with a fluoropyrimidine, oxaliplatin and irinotecan (July 2017; CheckMate 142) [52]; nivolumab and ipilimumab after a fluoropyrimidine, oxaliplatin and irinotecan (July 2018; CheckMate 142) [53]; and pembrolizumab as the first frontline approval in MSI-H/dMMR colorectal cancer (June 2020; KEYNOTE-177) [54]. While the tumor site-agnostic approval was groundbreaking, recently published data from KEYNOTE-158 showed a wide range of response rates to pembrolizumab in this setting based on primary tumor site [51], with the highest responses seen in endometrial cancer (57%) and the lowest with pancreatic (18%) and central nervous system (0%) malignancies. While larger cohorts and further study is needed into the molecular mechanisms of response [55], these findings suggest the site-agnostic model of ICI use should be evaluated thoughtfully in these and other MSI-H/dMMR solid tumors when other treatment options exist [56].

### 2.2. NTRK1/2/3 Fusions

*NRTK* fusions occur in <0.5% of all cancer types, but are enhanced in some rare cancers or those with atypical histology, including salivary carcinoma (5%), thyroid (2%), sarcoma (including uterine, 1%) and possibly glioblastoma multiforme (<1%) [57]. The FDA approved larotrectinib [15] on 26 November 2018 and entrectinib [16] on 15 August 2019 for adult and pediatric solid tumor patients whose metastatic or unresectable tumors do not contain a resistance mutation to either agent at the start of treatment. Approval of larotrectinib was based upon the efficacy observed in the first 55 patients of three multicenter, open-label, single-arm clinical trials (LOXO-TRK-14001, SCOUT, NAVIGATE), where Drilon et al. reported a response rate of 75%, which was ongoing in 71% of responders at one year [58]. Comprising a cohort of 17 different tumor types, patients harboring a TRK fusion and treated with larotrectinib primarily had grade 1 toxicities including increased liver enzymes, fatigue, nausea, vomiting and anemia. In a recent pooled analysis of a larger cohort from the same three trials, an ORR was found to be 79% for 153 evaluable patients, with 16% complete responses and a median duration of response of over 35 months [59]. Additionally, long-term toxicity data showed the safety of larotrectinib with no treatment-related deaths observed and the most frequent grade 3 or 4 toxicities were increased alanine aminotransferase levels (3%), neutropenia (2%) and anemia (2%).

The efficacy of entrectinib for patients with advanced solid tumors with *NTRK* fusions was likewise recently described in a combined analysis from three ongoing phase 1 and 2 clinical trials (ALKA-372-001, STARTRK-1 and STARTRK-2). Doebele and colleagues described a 57% response rate with a median duration of 10 months in a cohort of 54 patients (>60 had received prior systemic therapy) comprising 19 different histologies [60]. It should also be noted that in addition to activity against *NTRK* fusions, entrectinib is also FDA approved for the treatment of ROS proto-oncogene 1 (*ROS1*) rearranged NSCLC.

The robust efficacy of larotrectinib and entrectinib with a tolerable safety profile makes both agents attractive for *NTRK* fusion-positive metastatic cancer patients. Upon progression, molecular profiling should be repeated to assess for resistance mechanisms, as *NTRK* kinase domain mutations (involving solvent front, gatekeeper residue or xDFG motif) or off-target mutations (including Kirsten rat sarcoma viral oncogene homolog (*KRAS)* mutations, mesenchymal-epithelial transition (*MET)* amplifications and serine-threonine protein kinase B-RAF (*BRAF)* V600E mutations) can inform the next systemic approach [61]. If progression is restricted to a limited number of sites (termed oligometastatic), local therapy with continued *NTRK* inhibition can be pursued. However, if extensive progression occurs and *NTRK* resistance mutations are identified, next-generation TKIs repotrectinib [62,63] and seltrectinib [64] are currently being evaluated and show preliminary efficacy in this setting.

### 2.3. Tumor Mutational Burden

On 16 June 2020, the FDA approved pembrolizumab for its second tumor site-agnostic indication. Specifically, pembrolizumab can be considered following progression on another treatment for all metastatic or unresectable adult and pediatric solid tumor patients found to have high tumor mutational burden (TMB-H), defined as ≥10 mutations/megabase (mut/Mb). As a part of this approval, the FoundationOne CDx assay (Foundation Medicine, Cambridge, MA, USA) was authorized as a companion diagnostic for tissue TMB evaluation [14]. The approval was based upon recently published prospective data from 10 treatment-refractory cohorts in the phase 2 KEYNOTE-158 trial, which showed an ORR of 29% of 102 TMB-H patients to pembrolizumab, compared to 6% of those with TMB <10 mut/Mb [65].

The pan-tumor approval for TMB-H has been controversial in the oncology community. On one hand, some point to potential flaws in the study including a perceived arbitrary TMB cutoff of 10 mut/Mb, the lack of a control arm in KEYNOTE-158 and the composition of tumor types included in the 102 TMB-H patients (i.e., >60% were small cell lung, endometrial and cervical, which already have ICIs approved in some capacity; less commonly enrolled tumor types need a higher sample size to determine efficacy) [66]. To this end, McGrail and colleagues recently published retrospective data from The Cancer Genome Atlas (TCGA) which suggests a blanket TMB cutoff of 10 mut/Mb for pembrolizumab us may not be applicable in all solid tumor types. The authors first divided solid tumor patients into two categories, those with a positive versus no correlation between CD8 T-cell levels and neoantigen load, a term referring to the presumption that higher amounts of tumor mutations lead to certain antigen peptides which in turn activate the immune system. While TMB-H predicted a response to ICI in those with a correlation between CD8 T-cells and neoadjuvant load (e.g., melanoma, bladder and lung cancer), this benefit was not seen in those tumor types where no such correlation was seen (e.g., breast, prostate, glioma). In fact, the ORR for TMB-H patients in this latter subgroup was <20%, and TMB-H patients were actually found to have a lower ORR than those with low TMB (odds ratio (OR) = 0.46, 95% CI 0.24–0.88, *p* = 0.02) [67].

In contrast, advocates of utilizing TMB-H such as Subbiah and colleagues cite the durability of responses seen in KEYNOTE-158 (approximately half were for at least two years), the resultant expansion of genomic profiling to include rare tumors, improvement of pembrolizumab reimbursement and access given FDA approval, access for minority and underserved populations and overall enabling of patients and physicians to make informed clinical decisions [68]. As the careful use of TMB-H makes its way to the clinic, emerging co-occurring biomarkers, such as mutations in DNA polymerase epsilon (*POLE*) and delta 1 (*POLD1*) may help predict survival with ICI use [69], though no FDA approvals have yet been based on these alterations.

### 2.4. On the Horizon: PD-L1 Amplification

While requiring further study, additional biomarkers obtained from comprehensive molecular profiling may eventually be considered for predictive pan-tumor use. One such biomarker includes amplification of *PD-L1* (or *CD274*), which Goodman et al. identified in 0.7% of 118,000 profiled solid tumor patients and may predict efficacy to ICI [70]. This is in contrast to PD-L1 expression measured using immunohistochemistry, which is both more common and less predictive (see PD-L1 section below). With only limited published case reports or series to date [71,72,73], there does appear to be a histologic-dependent range in frequency of *PD-L1* amplifications with increased incidence in uncommon tumor types including bladder squamous cell, renal sarcomatoid, liver mixed hepatocellular and anaplastic thyroid carcinoma (all >5%) [70].

## 3. Molecular Alterations with Approvals in Multiple Tumor Types

While not approved in a site-agnostic fashion, a growing compendium of molecular biomarkers are susceptible to precision oncology therapies with FDA approval in more than one tumor type. The latter part of Table 1 provides a summary of the agents which are discussed below.

### 3.1. BRAF V600 Mutations

#### 3.1.1. Melanoma

Mutations in the 600th amino acid position of *BRAF* activate the mitogen-activated protein (MAP) kinase pathway, leading to cancer cell growth and proliferation. These lesions, predominantly V600E or V600K, can be identified in multiple solid tumors using NGS or other sequencing panels and are targetable. The prototype for targeting *BRAF* V600 lesions is cutaneous melanoma, where BRAF mutations occur in 40–60% and the single-agent selective BRAF-inhibitor, vemurafenib, was approved a decade ago [74]. Shortly following the FDA approval of vemurafenib, the single-agent BRAF-inhibitor dabrafenib [75] and the single-agent mitogen-activated protein kinase (*MEK*)-inhibitor trametinib [76] likewise showed activity, including activity against brain metastases in the case of dabrafenib [77], and both received approval in 2013. Due to the superior efficacy of alternative combination regimens and ICIs (see below), single-agent BRAF inhibition is currently given only if other agents are contraindicated while trametinib monotherapy is no longer recommended in BRAF mutant melanoma.

Predictable resistance to single-agent *BRAF* V600 inhibitors occurs through various means of reactivation of the MAP kinase pathway including paradoxical activation of downstream MEK [78]. Therefore, dual pathway blockade with both a BRAF and a MEK inhibitor has since become the predominant targeted approach to BRAF-mutant melanoma. First-line phase 3 trials comparing BRAF/MEK combinations to single-agent targeted therapy have shown an overall survival (OS) advantage for dual therapy and have led to the FDA approvals of dabrafenib and trametinib in 2014 (COMBI-d, COMBI-v) [79,80,81,82,83], vemurafenib and cobimetinib in 2015 (CO-BRIM) [84,85] and encorafenib and binimetinib in 2018 (COLUMBUS) [86,87,88]. For example, long-term follow-up of encorafenib and binimetinib showed a 34-month median OS with this combination versus 17 months with vemurafenib, amounting to a 39% decreased risk of death (HR 0.61; 95% CI, 0.48–0.79) [88]. While all three combinations are FDA approved and have ORRs of approximately 60–70% (vs. 50% for single-agent), median PFS of 11–15 months (vs. 7–10 months), the combination of encorafenib and binimetinib may be more tolerable with reduced pyrexia, fatigue and other symptoms [89].

It should also be noted that dabrafenib and trametinib were FDA approved in 2018 for the adjuvant treatment of *BRAF* V600E or V600K mutations following resection, which was based upon the COMBI-AD phase 3 study [90]. This represents one of the first approvals of a targeted agent for early-stage disease following surgery (with the exception of HER2 therapy for breast cancer; see below). Recently, Dummer et al. showed 5-year follow-up data, noting a 49% reduction for relapse or death for patients treated with planned 12 months of adjuvant dabrafenib and trametinib versus placebo [91].

Advances in targeted therapy for BRAF-mutated melanoma occurred at the same time as the development of ICI. In light of this, several additional points should be made in the management of BRAF-mutant melanoma. First, while *BRAF* V600 mutations predict response to targeted therapy, patients are also eligible to receive ICI in either the adjuvant [92,93] or metastatic [94,95,96,97,98] setting regardless of *BRAF* mutation status. Additionally, the IMspire150 trial was recently published, leading to the approval of the combination of agent atezolizumab (anti-PD-L1) and the BRAF/MEK inhibitors vemurafenib and cobimetinib in first-line metastatic disease [99]. The decision of whether to treat metastatic patients with ICIs, anti-BRAF/MEK agents or a combination of the two is not standardized, but involves shared-decision making with the patient and considerations such as toxicity differences, disease aggressiveness and pace, metastatic distribution including brain metastases, lactate dehydrogenase level and other clinical factors. Finally, it should be noted that only appropriate *BRAF* mutations (namely V600, with rare exceptions) are selected for targeted therapy, as utilization of BRAF/MEK inhibitors for certain non-V600 (e.g., K601E) lesions can paradoxically activate the MAP kinase pathway and possibly result in poor outcomes [100,101,102].

#### 3.1.2. Lung Cancer

In June 2017, the FDA approved the combination of dabrafenib and trametinib for metastatic NSCLC patients harboring *BRAF* V600E mutations based upon the international, multicenter, three-cohort, non-randomized, open-label BRF113928 trial [103,104,105]. Overall, 93 metastatic NSCLC patients were treated, with 36 receiving no prior therapy. Previously untreated patients showed an ORR of 64% with the majority partial responses; 69% had at least one grade 3–4 adverse event, including pyrexia, hypertension, increase in alanine aminotransferase and vomiting [105]. Subsequent molecular profiling of new tissue or liquid samples after progression has been described to show resistance mechanisms in MAP kinase signaling, such as MEK, KRAS and NRAS mutations [106].

#### 3.1.3. Thyroid Cancer

*BRAF* V600E mutations are frequent in differentiated thyroid cancer, occurring in almost 50% of papillary disease and associated with poor prognosis, especially when co-occurring with *TERT* promoter mutations [107,108]. For metastatic differentiated thyroid cancer patients harboring *BRAF* V600E mutations who are refractory to radioactive iodine, targeted therapy with BRAF-inhibitors dabrafenib or vemurafenib can be considered [109,110]. Anaplastic thyroid carcinoma is typically highly aggressive with a 1-year survival of roughly 20% [111]. Harboring *BRAF* V600 mutations in roughly 20–50% [112], Subbiah et al. showed a promising ORR of 69% (11 of 16) with dabrafenib and trametinib [113], ultimately leading to FDA approval of this BRAF/MEK combination for unresectable anaplastic thyroid carcinoma in 2018.

#### 3.1.4. Colorectal Cancer

Finally, on 8 April 2020, the FDA approved the doublet encorafenib and cetuximab for metastatic colorectal cancer containing a *BRAF* V600E mutation after receipt of prior therapy [114]. The approval was based on data from the randomized, phase 3 BEACON CRC trial, where both the triplet (encorafenib, cetuximab and MEK inhibitor binimetinib) and doublet (encorafenib plus cetuximab) arms similarly improved ORR, PFS and OS versus standard-of-care cetuximab plus irinotecan-based regimens. Updated analysis showed a similar median PFS of 4.5 months for the triplet arm and 4.3 months for the doublet arm, both superior to 1.5 months for control [115]. With similar efficacy and reduced toxicity including gastrointestinal and hematologic compared to the triplet, preference for FDA approval was given to doublet encorafenib and cetuximab in advanced *BRAF* V600E colorectal cancer.

### 3.2. ERBB2/HER2

#### 3.2.1. Breast and Gastric Cancer

Up to 20% of breast [116] and 13% of gastric cancers [117] overexpress the HER2 protein. That HER2 expression is a negative prognostic factor in breast and gastric cancer has been long recognized [118,119]. Generally, HER2 overexpression is determined by IHC testing (defined as 3+) and confirmed by reflex in-situ hybridization (ISH) testing for tumors with equivocal (2+) IHC results. Historically, tumors with 0 or 1+ expression by IHC have been denoted HER2-negative. Since 1998, trastuzumab, a monoclonal antibody targeting the HER2 protein has been available for the treatment of metastatic disease based on a number of studies [7,120,121,122]. Afterward, HER2 therapy with trastuzumab expanded into the adjuvant and neoadjuvant settings [123,124,125].

Numerous other HER-2 directed agents are now available. For example, in the metastatic setting, lapatinib was approved by the FDA in 2007 in combination with capecitabine and also has been shown to provide a benefit when combined with trastuzumab [126,127]. Additional monoclonal antibodies (pertuzumab, based on the CLEOPATRA study) [128] as well as ADCs (ado-trastuzumab emtansine (T-DM1) based on the EMILIA study, and fam-trastuzumab deruxtecan (T-DXd) based on the DESTINY-Breast01 study) [129,130] have entered practice in the metastatic setting either alone or in combination with trastuzumab. Additional TKIs (neratinib based on the NALA trial and tucatinib based on the HER2CLIMB study) [131,132] have also been approved. Pertuzumab can be effective when combined with trastuzumab and chemotherapy in adjuvant and neoadjuvant settings [133,134]. As a whole, advances in targeted agents with improved efficacy, including superior intracranial activity [135], have greatly improved the prognosis for breast cancer expressing HER2.

In gastric cancer, trastuzumab was initially approved in the metastatic setting in 2010 based on the TOGA trial [136]. Most recently, T-DXd received FDA approval in the metastatic setting in January 2021 based on the DESTINY-gastric01 trial [137]. In this latter study, T-DXd was compared to chemotherapy for metastatic patients who had received two prior systemic lines of therapy, including trastuzumab. Both an improvement in ORR (51% vs. 14%, *p* < 0.001) and median OS were seen (12.5 vs. 8.4 months, HR 0.59). Approximately 10% of T-DXd treated patients developed interstitial lung disease or pneumonitis, although the majority were grades 1 or 2.

#### 3.2.2. On-the Horizon: Colorectal Cancer

While no FDA approvals have occurred to date, several clinical trials have evaluated HER2-directed agents, either alone or in combination, for advances colorectal cancer patients exhibiting HER2 overexpression. Combinatorial regimens include pertuzumab and trastuzumab in the MyPathway and TRIUMPH trials [138,139]; trastuzumab and lapatinib in HERACLES-A [140]; trastuzumab and tucatinib in MOUNTAINEER [141]; and T-DM1 and pertuzumab in HERACLES-B [142]. Additionally, data for T-DXd in DESTINY-CRC01 was recently reported [143] Taken as a whole, these studies showed variable ORRs from 9% to 55% with seemingly better responses found for those patients whose tumors are KRAS wild-type.

#### 3.2.3. On-the-Horizon: HER2 Mutations in NSCLC

In addition to HER2 overexpression discussed in breast, gastric and colorectal cancer, activating HER2 mutations are found in multiple solid tumors and are potentially drug-able [144], but no approvals have occurred to date. In NSCLC, driver HER2 mutations are identified in approximately 2% of patients. In a phase II basket trial utilizing T-DM1 in mostly pre-treated metastatic NSCLC patients, 8/18 (44%) obtain a partial response [145]. Responses were seen in patients with HER2 exon 20 insertions as well as other kinase and non-kinase domain mutations. Additionally, Smit and colleagues reported data from the cohort of DESTINY-Lung01 which evaluated T-DXd in NSCLC patients with activating HER2 mutations, on which 90% were relegated to the kinase domain [146] With most patients previously receiving both chemotherapy and ICI, an impressive ORR of 62% (26/42) was seen, for which the median duration was not reached at the time of data cutoff (median follow-up eight months).

### 3.3. RET

#### Medullary Thyroid, Other Thyroid and Lung Cancers

An additional predictive molecular target with multi-tumor approved agents are *RET* alterations, with potentially sensitizing mutations seen in approximately 70% of medullary thyroid cancers (MTC), fusions in <10% of other thyroid cancers and fusions in 1–2% of NSCLC [147,148,149]. While ‘dirty’ multikinase inhibitors previously showed some activity in *RET*-altered disease, a tradeoff of significant toxicity was seen. However, in May 2020 the FDA approved selpercatinib, a selective small-molecule *RET* inhibitor for adult NSCLC patients with metastatic *RET* fusions, as well as patients ≥12 years old with *RET*-mutated MTC or fusion-positive thyroid cancer who require systemic therapy (and are refractory to radioactive iodine, if indicated). Approval was based upon the results of LIBRETTO-001, a multicenter, open-label, multi-cohort trial. Key findings reported in *RET* fusion-positive NSCLC include a 64% ORR of 105 consecutively enrolled and pre-treated patients, with a median duration of almost 18 months; for untreated NSCLC patients, an 85% ORR was seen [150]. Importantly, only 2% discontinued selpercatinib due to drug-related toxicity, and 91% (n = 11 of 12) with measurable central nervous system disease had an intracranial response. In *RET*-altered thyroid cancer, Wirth et al. detailed an ORR of 73% and 92% 1-year PFS of 88 untreated MTC patients harboring *RET* mutations; of 55 other MTC patients who received prior multi-kinase TKIs vandetinib and/or cabozantinib, impressive response rates and durability were still seen [151].

It should also be noted that pralsetinib was subsequently approved for *RET* fusions in advanced NSCLC in September 2020 and advanced, mutated or fusion-positive thyroid cancer patients (similar to the selpercatinib indication) in December 2020 based upon the ARROW trial. For NSCLC, a 57% ORR of 87 previously-treated patients was observed; further, an additional 27 untreated fusion-positive NSCLC patients had a 70% ORR to pralsetinib, with nearly 60% of responses extending beyond six months [37,152,153]. Clinical progression to selpercatinib (or pralsetinib) should prompt molecular testing for resistance mechanisms, which can include *RET* solvent front mutations, as well as *MET* and *KRAS* amplifications [154,155]. While some resistance mechanisms may be targetable, development of next-generation *RET* inhibitors is warranted for solvent front alterations.

### 3.4. DNA Damage Response/PARP Inhibition

Many loss-of-function alterations occur in genes involved in DNA repair, particularly in homologous recombination repair (HRR). BRCA1 and BRCA2 are the most common and most well-characterized genes involved in this process, but other genes such as ATM, CHEK2, PALB2 and RAD51 are also linked to HRR deficiency (HRD). Deleterious mutations in HRR can be found both in the germline setting as well as somatic alterations uncovered with tumor molecular profiling. Responses to poly(adenosine diphosphate-ribose) polymerase (PARP) inhibition have been described in several tumor types with HRD, either by specific mutations identified in a candidate gene or via genomic instability identified during molecular profiling.

#### 3.4.1. Ovarian Cancer

Ovarian cancer is known to be associated with germline alterations in BRCA1 or BRCA2, though somatic mutations can also occur. Olaparib was approved in 2018 for the maintenance of patients with somatic mutations in the setting of complete or partial response to first-line platinum therapy. This indication is based on the SOLO-1 trial comparing olaparib versus placebo in this setting. Progression-free survival was improved in the olaparib arm (HR 0.30; *p* < 0.0001) [156]. The indication was expanded to include a combination of olaparib with bevacizumab in 2020 based on the PAOLA-1 trial [157]. The PARP inhibitor rucaparib initially had approval only in patients with BRCA germline mutations. However, soon thereafter rucaparib was approved in the maintenance setting for ovarian cancer regardless of BRCA status. Interestingly, the PARP inhibitor rucaparib was later approved regardless of BRCA status based on the ARIEL3 trial. While this trial evaluated patients for BRCA or HRD, the overall study population showed a benefit in PFS (HR 0.36, *p* < 0.0001) [158]. In 2017, approval for the PARP inhibitor niraparib was obtained for maintenance therapy in ovarian cancer, also without the need for HRD mutations with this indication expanded to the first-line maintenance setting in 2020 based on the PRIMA trial [159]. In 2019, however, niraparib was approved again in late-line ovarian cancer for patients with HRD mutations [42]. As is clear from these approval timelines, PARP inhibitor therapy in ovarian cancer remains complex, with certain agents approved in certain settings regardless of genomic alterations, some approved only for BRCA1/2 genomic alterations and others approved for germline or somatic alterations in BRCA1/2 or HRD tumors.

#### 3.4.2. Prostate Cancer

The approval of PARP inhibitors for somatic HRD mutations in prostate cancer was based on the PROfound study which randomized 256 patients to the PARP inhibitor olaparib and 131 patients to the investigator’s choice of hormone therapy. Patients were divided into two cohorts, one with mutations in *BRCA1, BRCA2* or *ATM* and the other with mutations among 12 other genes in the HRR pathway. A statistically significant difference was seen in PFS among the BRCA/ATM cohort (HR 0.34, *p* < 0.0001) as well as overall across both cohorts (HR 0.49; *p* < 0.0001) [160]. Even more recently, the PARP inhibitor rucaparib was also approved in castrate-resistant prostate cancer in patients specifically with BRCA1 or two mutations, whether germline or somatic, who have failed hormone therapy and a taxane. The approval was based on the TRITON2 trial, a single-arm trial of 115 patients showing a confirmed objective response rate of 44% among the 65 patients who had measurable disease [161].

#### 3.4.3. Breast Cancer

In January 2018, the FDA approved olaparib in metastatic HER-2 negative breast cancer patients whose tumors harbored a germline *BRCA* mutation and received prior chemotherapy based upon the results of the randomized phase 3 OlympiAD trial [162]. In this study, olaparib was found to have an ORR of 60% and a superior PFS compared to physician’s choice of chemotherapy (7.0 versus 3.2 months; HR 0.58). Subsequently, talazoparib received a similar approval for germline *BRCA* mutated metastatic breast cancer, with the EMBRACA trial demonstrating a higher ORR (63% versus 27%, *p* < 0.001) and improved median PFS (8.6 versus 5.6 months; HR 0.54) when compared to chemotherapy [163].

#### 3.4.4. Pancreatic Cancer

Germline BRCA1/2 mutations occur in approximately 5% of pancreatic cancers [164]. On 27 December 2019, olaparib was FDA approved for this subset of metastatic pancreatic cancer patients in the maintenance setting, where the agent was given after a minimum of 16 weeks of platinum-based chemotherapy. Golan and colleagues reported an improvement in median PFS (7.4 versus 3.8 months for placebo; HR 0.53); however, an interim analysis did not show a difference in OS [165].

A summary of major clinical trials leading to approvals in more than one malignancy can be seen in Table 2.

### 3.5. PD-L1

The use of ICI therapy, particularly anti-programmed death-1 (PD1) antibodies or anti-programmed death ligand-1 (PD-L1) antibodies, has become virtually ubiquitous in cancer. Currently, there are seven anti-PD-1 or PD-L1 therapies in use in the clinic, approved across 19 malignancies and with 77 different indications [166,167,168,169,170,171,172]. Being as the mechanism of action of these therapies depends upon the interaction between PD-1 and PD-L1, PD-L1 protein expression was early determined to be a potential predictor of response to ICI [173]. However, the fact that some tumors with high expression of PD-L1 do not respond to PD-(L)1 therapy, and the fact that some tumors with no or low PD-L1 expression do respond to PD-(L)1 therapy, highlights the difficulty in using this biomarker as a true surrogate of response [173]. Additionally, various companion diagnostics using different detection antibodies, methods of measuring PD-L1 expression and “positive” cutoffs have been problematic and present a barrier to the interpretation of biomarker data in clinical trials [174]. In an evaluation of the pivotal trials leading to 45 FDA approvals of PD-(L)1 inhibitors from 2015–2019, PD-L1 expression was predictive in only 29% of the approvals, while it was not predictive in 53% and not tested in 18% [175]. However, while PD-L1 expression is not always predictive, failure to include the biomarker into certain clinical trials or utilization of the wrong assay or wrong cutoff may have led to a determination of an overall lack of efficacy [176,177]. At the current time, 12 indications for PD-(L)1 therapy in seven malignancies are dependent on PD-L1 status [166,167,168,171]. These indications utilize different methods of determining PD-L1 status (tumor cell proportion score, immune cell proportion score or combined positive score), different thresholds for positivity and different FDA-approved companion diagnostics. Table 3 summarizes the PD-L1 based approvals that exist in lung, head and neck, bladder, gastric, esophageal, cervical and breast cancer together with the various measures of PD-L1 expression, companion diagnostics and positive thresholds. In summary, PD-L1 remains a highly imperfect biomarker, and other markers of immune responsiveness are simultaneously being tested for (e.g., MSI status, TMB-h) and studied (tumor-infiltrating lymphocytes, tumor microenvironment, etc.) to enable proper selection of treatment with ICIs [174].

### 3.6. On the Horizon—KRAS G12C

*KRAS*, which controls cellular signal transduction through its encoded guanosine triphosphatase activity, is the most commonly mutated oncogene in solid tumors, frequently portends a poor prognosis, is affiliated with resistance to multiple systemic treatments and thus far, its targeting has remained elusive [189,190,191,192]. Occurring in approximately 13% of NSCLC and >1% of colorectal cancer and various other solid tumors, *KRAS* G12C mutations were found to be targetable in pre-clinical studies through irreversible, covalent binding of small molecule kinase inhibitors to the mutated cysteine and nearby P2 pocket of the switch II region [193,194,195]. Hong et al. recently reported phase 1 data, showing promising activity of *KRAS* G12C inhibitor, sotorasib, in 129 pre-treated (median number of prior lines of therapy was 3) solid tumor patients [196]. For example, 32% of NSCLC, 7% of colorectal cancer and 14% of other solid tumor patients (including melanoma, endometrial, pancreatic and appendiceal) had an objective response. While the median PFS of responders was six months with single-agent sotorasib, future evaluation of *KRAS* inhibitors with tumor-informed precision combinations may lead to more effective targeting [197,198,199]. Furthermore, the efficacy of a second covalent inhibitor of *KRAS* G12C, adagrasib, was presented at the European Lung Cancer Virtual Conference in early 2021, where the multi-cohort phase I/II trial showed a 45% ORR in 51 advanced, typically pre-treated NSCLC patients [200].

## 4. Precision Oncology in Lung Cancer

Perhaps more than any other solid tumor, patients with lung cancer can derive benefit from therapeutic options exposed following comprehensive molecular profiling. The leading cause of cancer-related mortality worldwide [201], advanced lung cancer treated with a one-size-fits-all approach of platinum doublet chemotherapy historically resulted in relatively poor outcomes, with a limited percentage of patients achieving long-term survival [202]. However, recent evidence showed a population-level reduction in mortality of lung cancer patients from 2013–2016, attributed not only to a reduction in incidence but an early indication of the efficacy of novel precision oncology treatments in NSCLC [203].

NSCLC accounts for approximately 85% of all lung cancer; its three major histologic types include adenocarcinoma, squamous cell carcinoma and large cell carcinoma. The vast majority of lung adenocarcinoma is driven by identifiable oncogenic aberrations, with a growing number amenable to targeted therapies such that current guidelines recommend complete molecular testing for patients with metastatic disease [204]. Additionally, testing should be considered for non-adenocarcinoma NSCLC, especially those with limited smoking history or mixed histology whose samples may be enriched for targetable mutations or alterations. While testing may take several forms, up-front comprehensive molecular profiling in NSCLC should ideally consist of a broad-panel evaluation such as NGS for specific gene mutations, IHC, ISH, real-time PCR and RNA assessment to identify gene rearrangements and fusions, as well as immune biomarker appraisal with IHC for PD-L1, MSI status and TMB (see previous discussion) [205]. If available tissue is not sufficient to complete testing, liquid biopsy with plasma cell-free or circulating tumor DNA can be informative [206,207]. Unless an impending clinical scenario mandates, the treatment team should ideally wait upon receipt of comprehensive molecular data to determine if the NSCLC patient is a candidate for first-line targeted therapy. The determination of whether to begin first-line treatment with ICI or targeted therapy is of great importance, as improper initial ICI in NSCLC patients with specific oncogenic drivers can lead to significant toxicity when subsequent TKIs or targeted therapy are begun [208,209]. In addition to the aforementioned targetable alterations (BRAF V600E, *RET* fusions, as well as emerging *HER2* mutations and *KRAS* G12C), multiple other predictive molecular targets exist in NSCLC. Table 4 shows molecular alterations and their targeted agents that have been approved only in lung cancer.

### 4.1. EGFR

Epidermal growth factor receptor (*EGFR*) is mutated in approximately 10% of Caucasian and potentially up to 50% of Asian NSCLC patients with limited or no smoking history [225]. The majority of *EGFR* mutations sensitive to targeted therapy lie within the tyrosine kinase domain, with exon 19 deletions or exon 21′s L858R comprising the vast majority. Other rare lesions may also be sensitive to *EGFR* TKIs and include L861Q, G719X and S768I [226]. Current oral TKIs with FDA approved to treat advanced NSCLC patients whose tumors harbor sensitizing *EGFR* mutations include gefitinib, erlotinib (with or without ramucirumab), afatinib, dacomitinib and osimertinib.

Initially, first-generation oral TKIs gefitinib and erlotinib showed promising activity for inhibition of sensitizing *EGFR* mutations. In the randomized phase 3 trial, IPASS, first-line gefitinib was affiliated with an improved response rate versus platinum-based doublet chemotherapy (71% vs. 43%) in Asian patients with *EGFR* mutations [227]. While this agent also showed a prolonged PFS, subsequent results did not translate into an improved OS, likely due to ensuing TKI use of the chemotherapy arm upon progression [228]. These findings were also confirmed in Caucasian NSCLC patients with *EGFR* mutations [229]. Likewise, EURTAC detailed a prolonged PFS for first-line erlotinib versus chemotherapy in European *EGFR*-mutated NSCLC patients (9.7 vs. 5.2 months; HR 0.37) [230]. FDA approval was also granted in 2020 to the combination of ramucirumab, a recombinant monoclonal antibody targeting vascular endothelial growth factor (VEGF) receptors, plus erlotinib for first-line use in metastatic NSCLC patients with exon 19 deletions or L858R mutations. Approval was granted based on results of a randomized phase 3 trial, RELAY, which showed a prolonged PFS for the combination versus erlotinib monotherapy (19.4 vs. 12.4 months; HR 0.59); a similar response rate of both arms was observed (approximately 75%) [231]. However, 72% of patients in the combination arm had grade 3–4 adverse events including hypertension and transaminase abnormalities; one treatment-related death in the combination arm occurred. While FDA approved and an option to be discussed with patients, the niche of combinatorial strategies of *EGFR* TKIs with VEGF receptor inhibitors (e.g., ramucirumab or bevacizumab) [232] in the frontline setting warrants further exploration, especially when next-generation TKIs (see osimertinib below) are very efficacious and have a favorable toxicity profile.

Second-generation TKIs include afatinib and dacomitinib, which irreversibly inhibit multiple ErbB/HER receptors, including EGFR. Phase 3 LUX-Lung 3 showed an improvement of PFS in advanced lung adenocarcinoma patients with sensitizing *EGFR* mutations treated with afatinib compared to cisplatin plus pemetrexed (11.1 vs. 6.9 months). Additionally, afatinib was FDA approved in 2018 for uncommon mutations S768I, L861Q and/or G719X based upon combined analysis from the LUX-Lung 2, 3 and 6 trials [233,234]. Dacomitinib received FDA approval in 2018 following published results from the randomized phase 3 ARCHER1050 study, with updated data showing a prolongation of OS versus gefitinib in the first-line setting (median 34.1 vs. 26.8 months; HR 0.76) [235,236].

Initially FDA approved in 2017 following progression on another TKI based on its efficacy in the AURA3 trial against *EGFR* resistance mutation, T790M [237], third-generation TKI osimertinib is now considered a standard-of-care for untreated, advanced NSCLC patients with sensitizing *EGFR* aberrations based on the FLAURA study [238]. Most recently, secondary endpoint OS analysis was published, showing a significant prolongation of median OS in the osimertinib arm compared to TKIs gefitinib or erlotinib (38.6 vs. 31.8 months; HR 0.80) [239]. The attraction of osimertinib includes not only its activity against T790M, but a relatively mild toxicity profile with QT prolongation (10%, reduced cardiac ejection fraction (5%), pneumonitis (2%) and interstitial lung disease (2%) with no treatment-related deaths. Further, osimertinib can induce durable intracranial responses, including in patients with leptomeningeal disease [240,241].

The clinical utility of osimertinib in *EGFR*-mutated NSCLC is extending to other indications. For example, the agent could be considered as a possible alternative to afatinib for advanced NSCLC patients whose tumor harbors uncommon *EGFR* mutations [242,243]. Additionally, the recently-published ADAURA trial [244] showed an impressive disease-free survival benefit at two years with the addition of osimertinib to provider-determined adjuvant therapy in stage II and IIIA patients (90% versus 44% for placebo; HR 0.17). Further, only 1% of patients receiving osimertinib developed CNS recurrence versus 10% of the placebo arm at two years. The adjuvant use of osimertinib was FDA approved on 18 December 2020 and extended to stage IB-IIIA NSCLC (non-squamous) patients whose tumors harbored EGFR exon 19 deletions or exon 21 L858R mutations [215]. This approval represents a paradigm shift, as increasing numbers of non-metastatic NSCLC patients will now receive molecular profiling whereas testing was previously relegated to the advanced setting. The additional genomic information received and potential uncovering of targetable molecular targets in the early-stage space provides a clinical challenge and opportunity for further study. Upon resistance to osimertinib, it is important to re-biopsy, if possible, and obtain tissue or liquid molecular profiling both to assess for small cell transformation, as well as potential targetable resistance mechanisms including *MET* amplifications, additional *EGFR* mutations or rare fusion events [245].

Finally, it should be noted that exon 20 insertion mutations represent the third most common alteration of *EGFR* and are generally not sensitive to TKIs. At this time, two agents selective to exon 20 insertions have received FDA Breakthrough Therapy Designation, amivantamab-vmjw [246] and mobocertinib [247]. On May 21, 2021, amivantamab, a bispecific antibody to EGFR and MET, was FDA approved for advanced NSCLC patients with *EGFR* exon 20 mutations after progression with chemotherapy. The approval was based upon the phase 1 CHRYSALIS study, which showed a 40% ORR with a median duration exceeding 11 months in 81 evaluated patients [246].

### 4.2. ALK, ROS1

Anaplastic lymphoma kinase (*ALK;* 2–5% of NSCLC) and *ROS1* (1–3%) rearrangements represent another subset of oncogenic drivers in NSCLC for which there are multiple effective targeted agents. Three precision drugs target both *ALK* and *ROS1* (crizotinib (which also has activity against *MET*) [248,249,250,251,252], ceritinib [253] and lorlatinib) [254,255]. *ALK* can also be inhibited by alectinib (which also has activity against *RET*) [256,257,258] and brigatinib [259,260,261,262], while *ROS1* is also inhibited by entrectinib (previously discussed for *NTRK* fusions) [263]. In the first-line setting for *ALK*-rearranged disease, current preference should be given to alectinib, brigatinib or lorlatinib over crizotinib, as all three agents showed improved efficacy in randomized phase 3 trials compared to the first-generation *ALK* inhibitor [255,256,257,258,261,262]. For example, updated data from the ALEX trial recently established an advantage of alectinib over crizotinib for mature PFS (median 34.8 vs. 10.9 months; HR 0.43) and median OS (not reached vs. 57.4 months; HR 0.67), which was also seen for patients with brain metastases [258]. While ceritinib remains an FDA-approved frontline option based on the ASCEND-4 trial [253], a direct comparison to crizotinib or another TKI has not been published to date.

The toxicity profile should be considered before the use of any TKI in the management of *ALK*-rearranged NSCLC, such as myalgia, edema, hepatotoxicity, interstitial lung disease/pneumonitis and bradycardia with alectinib and respiratory symptoms [264,265], vision change, amylase and lipase elevation, hypertension and similarly bradycardia with brigatinib. Although associated with undesirable toxicities including cognitive effects, mood changes, peripheral neuropathy and elevated triglycerides or cholesterol, lorlatinib is a third-generation TKI that has ample CNS penetration and has emerged as an effective agent with not only front-line activity, but efficacy at progression for multiple *ALK* resistance mutations from early-generation inhibitors [255,266,267,268]. As with *EGFR*, the mechanism of resistance to *ALK*-inhibition should be sought with repeat tissue or liquid biopsy as targetable resistance mechanisms aside from *ALK* mutations may be identified [269].

Of the three available agents that target *ROS1*, entrectinib and crizotinib are FDA approved in advanced ROS1-rearranged NSCLC and should be prioritized over ceritinib in the first-line setting. Further, in addition to pulmonary toxicity seen with almost all TKIs utilized in NSCLC, ceritinib use includes heightened gastrointestinal toxicities such as diarrhea, nausea and vomiting, hepatotoxicity and pancreatitis that may make it less tolerable than other agents [253]. Both entrectinib and crizotinib provide ORR of approximately 70–80%, a significant portion of which are durable [252,263]; further, intracranial response of entrectinib is reported as 55%.

Upon progression, lorlatinib has likewise emerged as a preferred agent subsequent to either crizotinib or entrectinib [270,271]. Next-generation TKIs such as repotrectinib, which has impressive CNS penetration and activity against *ROS1*, *ALK* and *NTRK*, are currently being evaluated for first-line or subsequent-line use [272].

### 4.3. MET

Alterations in oncogenic driver *MET* occur in at least 3–5% of NSCLC and classically are affiliated with poor prognosis. While not all mutations are susceptible to targeted therapy, tumors with *MET* exon 14 lesions or a significantly elevated gene copy number may predict efficacy to TKIs [273]. In particular, *MET* exon 14 alterations are sensitive to inhibition with multi-kinase inhibitors crizotinib [274], cabozantinib [275], as well as recently FDA-approved selective inhibitors capmatinib [276,277] and tepotinib [277]. The GEOMETRY mono-1 trial reported a 68% response rate with capmatinib for untreated patients whose NSCLC contained *MET* exon 14 skipping mutations with a 12.6-month median duration of response. The authors reported a 40% response rate first-line for those harboring *MET* amplifications with a gene copy number of at least 10 [278]. Toxicity from this next-generation TKI is relatively modest and most frequently includes peripheral edema and nausea. Likewise, the efficacy of tepotinib was evaluated in the VISION trial, where 152 advanced NSCLC patients with exon 14 skipping mutations showed a 43% ORR (the same ORR was seen for treatment-naïve or those previously treated), with a median response duration of approximately 11 months. Combined with reports of effective CNS activity, capmatinib [278] or tepotinib [279,280] should be considered as the first-line options utilized for *MET*-directed therapy.

In addition to FDA-approved agents targeting sensitive alterations in *EGFR*, *ALK*, *ROS1*, *BRAF*, *MET*, *RET* and *NTRK*, the precision therapeutic arsenal in NSCLC may soon expand to other oncogenic drivers, including previously-cited *KRAS* G12C (13% of all NSCLC) [196] and *HER2* exon 20 mutations [146] lending further credence to the necessity of molecular profiling in this target-rich disease.

## 5. Conclusions

As we have attempted to show, advances in molecular profiling have enabled genomic classification of a patient’s tumor, leading to the development, approval and availability of precision therapies including TKIs, ADCs and ICIs. With this ever-expanding arsenal of treatment options and increasing availability of next-generation sequencing, the utilization of molecular profiling is primed to expand to most advanced solid tumors into early-stage disease and include combinatorial precision regimens based on complex molecular findings [281]. It is incumbent for the modern oncologist to be well-versed regarding the range of potentially targetable aberrations available, be comfortable with a molecular profiling platform he or she trusts and be able to effectively interpret resultant data to help patients make informed decisions with regards to treatment.

## Figures and Tables

**Table 1:** Molecular alterations guiding therapy with agents approved in multiple tumor types.

Alteration(s)	Indication	Line of Therapy	Medications	Drug Class	FDA Approval Date
MSI-h/ dMMR	Any tumor type	>2nd line metastatic	Pembrolizumab	PD-1 mAb	23 May 2017 [9]
	Colorectal cancer	2nd line metastatic	Nivolumab	PD-1 mAb	31 July 2017 [10]
		2nd line metastatic	Ipilimumab + Nivolumab	CTLA-4 mAb + PD-1 mAb	10 July 2018 [11]
		1st line metastatic	Pembrolizumab	PD-1 mAb	29 June 2020 [12]
	Endometrial cancer	Progression after platinum	Dostarlimab	PD-1 mAb	22 April 2021 [13]
TMB-h (>10 mut/Mb)	Any tumor type	>2nd line metastatic	Pembrolizumab	PD-1 mAb	16 June 2020 [14]
NTRK fusions	Any tumor type	Any line	Larotrectinib	Trk inhibitor	26 November 2018 [15]
			Entrectinib	Trk inhibitor	15 August 2019 [16]
BRAF V600 mt	Melanoma	Any metastatic line	Vemurafenib ^1^	BRAF inhibitor	17 August 2011 [17]
			Dabrafenib ^1^	BRAF inhibitor	29 May 2013 [18]
			Trametinib ^1^	MEK inhibitor	29 May 2013 [18]
			Vemurafenib + Cobimetinib	BRAF + MEK	10 November 2015 [19]
			Dabrafenib + Trametinib	BRAF + MEK	9 January 2014 [20]
			Encorafenib + Binimetinib	BRAF + MEK	27 June 2018 [21]
		Adjuvant	Dabrafenib + Trametinib	BRAF + MEK	30 April 2018 [22]
		1st line	Atezolizumab, Vemurafenib and Cobimetinib	PD-L1 + BRAF + MEK	30 July 2020 [23]
	NSCLC	2nd line	Dabrafenib + Trametinib	BRAF + MEK	22 June 2017 [24]
	Anaplastic thyroid	Any line	Dabrafenib + trametinib	BRAF + MEK	4 May 2018 [25]
	Colorectal	2nd line	Encorafenib + cetuximab	BRAF inh + EGFR mAb	8 April 2020 [26]
HER2 (ERBB2) overexpression	Breast ^2^	Neoadjuvant, adjuvant or metastatic	Trastuzumab	Anti HER2 mAb	25 September 1998 [27]
			Pertuzumab	Anti HER2 mAb	8 June 2012 [28]
		Adjuvant or metastatic	Ado-trastuzumab emtansine (TDM-1)	Antibody drug conjugate	22 February 2013 [29]
			Neratinib	Small molecule	17 July 2017 [30]
		Metastatic	Fam-trastuzumab deruxtecan	Antibody drug conjugate	20 December 2019 [31]
			Lapatinib	Small molecule	13 March 2007 [32]
			Tucatinib	Small molecule	17 April 2020 [33]
	Gastric/GEJ	1st line metastatic	Trastuzumab	Anti HER2 mAb	20 October 2010 [34]
			Fam-trastuzumab deruxtecan	Antibody drug conjugate	15 Januray 2021 [35]
RET alterations	Medullary thyroid cancer (RET-mutated)	Metastatic	Selpercatinib	Small molecule RET inhibitor	8 May 2020 [36]
	Any thyroid cancer (RET fusion)	Metastatic	Selpercatinib		8 May 2020 [36]
	NSCLC (RET fusion)	Metastatic	Selpercatinib		8 May 2020 [36]
			Pralsetinib		4 September 2020 [37]
DNA repair deficiency *(Either BRCA1/2 somatic mutations, BRCA1/2 germline mutations, homologous repair deficiency (HRD) or homologous recombination repair mutations (HRR)*	Ovarian cancer	1st line or late-line maintenance	Olaparib (BRCA germline, somatic or HRD)	PARP inhibitor	19 December 2018 (BRCA), 8 May 2020 (HRD) [38]
			Rucaparib (initially for BRCA mutation, now regardless of biomarker status)	PARP inhibitor	19 December 2016 (BRCA) [39] 6 April 2018 (regardless of biomarker) [40]
			Niraparib (regardless of biomarker status for maintenance, late line for HRD)	PARP inhibitor	27 March 2017 (regardless of biomarker) [41] 10/23/2019 (HRD) [42]
	Breast cancer	>2nd line metastatic	Olaparib (BRCA germline only)	PARP inhibitor	12 January 2018 [43]
			Talazoparib (BRCA germline only)	PARP inhibitor	16 October 2018 [44]
	Pancreatic cancer	Metastatic maintenance	Olaparib (BRCA germline only)	PARP inhibitor	30 December 2019 [45]
	Prostate cancer	Metastatic	Rucaparib (BRCA germline or somatic)	PARP inhibitor	15 May 2020 [46]
			Olaparib (HRR germline or somatic)	PARP inhibitor	19 May 2020 [47]

^1^ No longer preferred as single agents in this disease. ^2^ Many HER2 agents may be used in combination with other HER2 agents or with chemotherapy, depending on the indication. Approvals given for HER2 agents are for the first approval of each drug.

**Table 2:** Key pivotal trials leading to FDA approval in alterations with multiple tumor indications (MSI-h, TMB-h, *NTRK* fusion, *BRAF*, *HER2, RET, BRCA*).

Setting/Genomic Alteration	Cancer Type, Line of Therapy	Study	Trial Phase	Number of Subjects	Line of Therapy	Agent	Comparator	Primary Outcome	Primary Outcome Results	Key Secondary Outcomes	Results (If Applicable)
MSI-h, dMMR	Pan-tumor	KEYNOTE-158 [51]	II	233	Metastatic 2nd line or greater	Pembrolizumab	None	ORR (objective response rate)	34.3% (95% CI, 28.3%, 40.8%)	Overall survival (OS)	23.5 mo (95% CI, 13.5-not reached (NR))
	Colorectal cancer	CheckMate 142 [53]	II	119	Metastatic any line	Nivolumab + Ipilimumab	None	ORR	55% (95% CI, 45.2–63.8%)	Disease control rate (DCR) >12 weeks	80%
		KEYNOTE-177 [54]	III	307	Metastatic 1st line	Pembrolizumab	Chemotherapy	Progression-free survival (PFS) (median)	16.5 v 8.2 months, hazard ratio (HR) 0.60 (95% CI, 0.45–0.8, *p* 0.0004)	ORR	44% vs. 33%
TMB-high	Pan-tumor	KEYNOTE-158 [65]	II	102	Metastatic 2nd line or greater	Pembrolizumab	TMB-low patients (n = 688)	ORR	29% (95% CI, 21–39%) vs. 6% (95% CI, 5–8%)	N/A	N/A
NTRK fusion	Pan-tumor	LOXO-TRK-14001, SCOUT, NAVIGATE (pooled analysis) [59]	I/II	159	Any metastatic	Larotrectinib	None	ORR	79% (95% CI, 72–85%)	Duration of response (DOR) (median)	35.2 mo (95% CI 22.8-NR)
		ALKA-372-001, STARTRK-1, STARTRK-2 (pooled analysis) [60]	I/II	54	Any metastatic	Entrectinib	None	ORR	57% (95% CI, 43–71%)	DOR (median)	10 mo (95% CI, 7.1-not estimable (NE))
BRAF V600E	Melanoma	COMBI-d [80]	III	423	Metastatic 1st line	Trametinib and Dabrafenib	Dabrafenib	PFS (median)	11.0 mo (95% CI, 8.0–13.9) vs. 8.8 mo (95% CI, 5.9–9.3)	OS (median)	25.1 v 18.7 months HR 0.71 (95% CI, 0.55–0.92, *p* = 0.01)
		CoBRIM [85]	III	495	Metastatic 1st line	Vemurafenib and Cobimetinib	Vemurafenib	PFS (median)	12.3 v 7.2 months HR 0.58 (95% CI, 0.46–0.72, *p* = < 0.0001)	OS (median)	22.3 v 17.4 months HR 0.70 (95% CI, 0.55–0.90, *p* = 0.005)
		COLUMBUS [87]	III	383	Metastatic 2nd line or greater	Encorafenib and Binimetinib	Vemurafenib	PFS (median)	14.9 v 7.3 months HR 0.54 (95% CI, 0.41–0.71, *p* <0.0001)	OS (median)	33.6 v 16.9 months HR 0.61 (95% CI, 0.48–0.79, *p* < 0.0001)
		COMBI-AD [90]	III	870	Stage III adjuvant	Dabrafenib and Trametinib	Placebo	Relapse-free survival (RFS) (three-year)	58% vs. 39% HR 0.47 (95% CI, 0.39–0.58, *p* < 0.001)	OS (three-year)	86% vs. 77%, HR 0.57 (95% CI, 0.42–0.79, *p* = 0.0006)
	NSCLC	BRF113928 [105]	II	36	Metastatic 1st line	Dabrafenib and Trametinib	None	ORR	64% (95% CI, 46–79%)	N/A	N/A
	Anaplastic thyroid cancer	CDRB436 × 2201 [113]	II	16	Any line post-radiation or surgery	Dabrafenib and Trametinib	None	ORR	69% (95% CI, 41–89%)	DOR (median)	Not reached
	Colorectal cancer	BEACON-CRC [114]	III	665	Metastatic 2nd line or greater	Encorafenib, Binimetinib and Cetuximab	Investigator choice	OS (median)	9.0 mo vs. 5.4 mo, HR 0.52 (95% CI, 0.39–0.70, *p* < 0.001)	ORR	26% (95% CI 18–35%) vs. 2% (95% CI, 0–7%)
HER2 positive	Breast cancer	Slamon et al. (2001) [7]	III	469	Metastatic 1st line	Trastuzumab and chemotherapy	Placebo and chemotherapy	PFS (median)	7.4 mo vs. 4.6 mo, HR 0.51 (95% CI, 0.41–0.63, *p* < 0.001)	OS (median)	25.1 mo vs. 20.3 mo, HR 0.80 (95% CI 0.64–1.00, *p* = 0.046)
		Geyer et al. (2006) [126]	III	399	Metastatic 2nd line or greater	Lapatinib and Capecitabine	Capecitabine	Time to progression (TTP) (median)	8.4mo vs. 4.4mo, HR 0.49 (95% CI, 0.34–0.71, *p* < 0.001)	N/A	N/A
		EMILIA [130]	III	991	Metastatic 2nd line or greater	Trastuzumab emtansine (T-DM1)	Lapatinib and Capecitabine	PFS (median)	9.6 mo vs. 6.4 mo, HR 0.65 (95% CI, 0.55–0.77, *p* < 0.001)	OS (median)	30.9 mo vs. 25.1 mo, HR 0.68 (95% CI, 0.55–0.85, *p* < 0.001)
		DESTINY-Breast01 [129]	II	184	Metastatic 3rd line or greater	Trastuzumab deruxtecan (T-DXd)	None	ORR	60.9% (95% CI, 53–68%)	PFS	16.4 mo (95% CI, 12.7-NR)
		CLEOPATRA [128]	III	808	Metastatic 1st line	Pertuzumab, trastuzumab and docetaxel	Trastuzumab and docetaxel	PFS (median)	18.5 mo vs. 12.4 mo, HR 0.62 (95% CI, 0.51–0.75, *p* < 0.001)	OS	HR 0.64 (95% CI, 0.47–0.88, *p* = 0.005)
		NALA [131]	III	621	Metastatic 2nd line or greater	Neratinib and capecitabine	Lapatinib and Capecitabine	PFS	HR 0.76 (95% CI 0.63–0.93, *p* = 0.0059)	OS (co-primary endpoint)	HR 0.88 (95% CI, 0.72–1.07, *p* = 0.21)
		HER2CLIMB [132]	II	612	Metastatic 3rd line or greater	Tucatinib, trastuzumab and capecitabine	Trastuzumab and capecitabine	PFS (median)	7.8 mo vs. 5.6 mo, HR 0.54 (95% CI, 0.42–0.71, *p* < 0.001)	OS (median)	21.9 mo vs. 17.4 mo, HR 0.66 (95% CI, 0.50–0.88, *p* = 0.005)
	Gastric cancer	TOGA [136]	III	594	Metastatic 1st line	Trastuzumab and Chemotherapy	Chemotherapy	OS (median)	13.8 mo vs. 11.1 mo, HR 0.74 (95% CI, 0.60–0.91, *p* = 0.0046)	N/A	N/A
		DESTINY-Gastric01 [137]	II	187	Metastatic 3rd line or greater	T-DXd	Chemotherapy	ORR	51% vs. 14% (*p* < 0.001)	OS (median)	12.5 mo vs. 8.4 mo, HR 0.59 (95% CI 0.39–0.88, *p* = 0.01)
RET fusion	NSCLC	LIBRETTO-001 [150]	I/II	105	Metastatic previously treated	Selpercatinib	None	ORR	64% (95% CI, 54–73%)	DOR (median)	17.5 mo (95% CI, 12.0-NE)
		ARROW [153]	I/II	87	Metastatic previously treated	Pralsetinib	None	ORR	57% (95% CI, 46–68%)	N/A	N/A
RET mutation	Medullary thyroid cancer	LIBRETTO-001 [151]	I/II	88	Metastatic 1st line	Selpercatinib	None	ORR	73% (95% CI, 62–82%	N/A	N/A
BRCA or HRD alteration	Ovarian cancer	SOLO-1 [156]	III	391	Metastatic 1st line maintenance	Olaparib	Placebo	PFS (-three-year)	60% vs. 27%, HR 0.30 (95% CI, 0.23–0.41, *p* < 0.001)	N/A	N/A
	Castrate-resistant prostate cancer	PROfound [160]	III	245	Metastatic	Olaparib	Enzalutamide or Abiraterone	PFS (median)	7.4 vs. 3.6 mo, HR 0.34 (95% CI, 0.25–0.47, *p* < 0.0001)	OS (median)	19.1 vs. 14.7 mo, HR 0.69 (95% CI, 0.50–0.97, *p* = 0.018)
		TRITON2 [161]	II	115	Metastatic, post androgen and chemotherapy	Rucaparib	None	ORR	43.5% (95% CI, 31.0–56.7%)	Prostate-specific antigen response	54.8% (95% CI 45.2–64.1%)
	Breast cancer	OlympiAD [162]	III	301	Metastatic, germline, no more than two prior lines	Olaparib	Chemotherapy	PFS (median)	7.0 vs. 4.2 mo, HR 0.58 (95% CI, 0.43–0.80, *p* = 0.0009)	OS (median)	19.3 vs. 17.1 mo, HR 0.90 (95% CI, 0.66–1.23)
	Pancreatic cancer	POLO [165]	III	154	Metastatic, germline, 1st line maintenance	Olaparib	Placebo	PFS (median)	7.4 vs. 3.8 mo, HR 0.53 (95% CI 0.35–0.81, *p* = 0.0035)	ORR	23% vs. 12%

**Table 3:** Indications for use of PD-1 or PD-L1 antibodies dependent on PD-L1 level.

Malignancy	Line	Agent	Measurement	Positive Threshold	FDA-Approved Companion Diagnostic	FDA Approval Date
**Lung cancer**	1st line metastatic	Pembrolizumab	Tumor proportion score (TPS)	>1%	22c3 Ab, Dako	11 March 2019 [178]
		Nivolumab + Ipilimumab	TPS	>1%	28–8 Ab, Dako	15 May 2020 [179]
		Atezolizumab	Tumor cell proportion score (TC)	>50%	SP142 Ab, Ventana	18 May 2020 [180]
			Immune cell proportion score (IC)	>10%	SP142 Ab, Ventana	
		Cemiplimab	TPS	>50%	22c3 Ab, Dako	22 February 2021 [181]
**Head and neck cancer**	1st line metastatic	Pembrolizumab	TC + IC (combined positive score or CPS)	>1	22c3 Ab, Dako	10 June 2019 [182]
**Bladder cancer**	1st line metastatic cisplatin ineligible	Pembrolizumab	CPS	>10	22c3 Ab, Dako	19 June 2018 [183]
		Atezolizumab	IC	>5%	SP142 Ab, Ventana	19 June 2018 [183]
**Gastric cancer**	>3rd line metastatic	Pembrolizumab	CPS	>1	22c3 Ab, Dako	22 September 2017 [184]
**Esophageal cancer (squamous)**	>3rd line metastatic	Pembrolizumab	CPS	>10	22c3 Ab, Dako	30 July 2019 [185]
**Cervical cancer**	>2nd line metastatic	Pembrolizumab	CPS	>1	22c3 Ab, Dako	12 June 2018 [186]
**Breast cancer (triple negative)**	Metastatic	Atezolizumab	IC	>1%	SP142 Ab, Ventana	8 March 2019 [187]
		Pembrolizumab	CPS	>10	22c3 Ab, Dako	13 November 2020 [188]

**Table 4:** Molecular alterations leading to FDA-targeted therapy approvals only in NSCLC.

Alteration(s)	Line of Therapy	Medications	Drug Class	FDA Approval Date
EGFR (exon 19 deletions and exon 21 point mutations)	1st line metastatic	Erlotinib	EGFR TKI	14 May 2013 [210]
		Gefitinib		13 July 2015 [211]
		Afatinib		12 January 2018 [212]
		Dacomitinib		27 September 2018 [213]
		Osimertinib (also against T790M mutations)		18 April 2018 [214]
	Adjuvant	Osimertinib (also against T790M mutations)		18 December 2020 [215]
EGFR (exon 20 insertion)	2nd line metastatic	Amivantamab	EGFR, MET bispecific antibody	21 May 2021 [216]
ALK fusions	Metastatic	Crizotinib	ALK TKI	26 August 2011 [217]
		Ceritinib		29 April 2014 [218]
		Lorlatinib		2 November 2018 [219]
		Alectinib		11 December 2015 [220]
		Brigatinib		28 April 2017 [221]
ROS1 fusions	Metastatic	Crizotinib	ALK TKI	11 March 2016 [222]
		Entrectinib	Selective TKI	15 August 2019 [16]
MET exon 14 skipping mutations	Metastatic	Capmatinib	MET inhibitor	6 May 2020 [223]
		Tepotinib		3 February 2021 [224]

## Data Availability

All data reported in this paper is available in the cited references, available either at pubmed.gov or at the websites within the citations.
